# Injuries Associated with Hoverboard Use: A Case Series of Emergency Department Patients

**DOI:** 10.5811/westjem.2017.6.34264

**Published:** 2017-09-22

**Authors:** Gregory S. Weingart, Lindsey Glueckert, Girlyn A. Cachaper, Kathie S. Zimbro, Ralitsa S. Maduro, Francis Counselman

**Affiliations:** * Eastern Virginia Medical School, Department of Emergency Medicine, Norfolk, Virginia; † Emergency Physicians of Tidewater, Virginia Beach, Virginia; ‡ Sentara Healthcare Quality Research Institute, Norfolk, Virginia

## Abstract

**Introduction:**

Since hoverboards became available in 2015, 2.5 million have been sold in the US. An increasing number of injuries related to their use have been reported, with limited data on associated injury patterns. We describe a case series of emergency department (ED) visits for hoverboard-related injuries.

**Methods:**

We performed a retrospective chart review on patients presenting to 10 EDs in southeastern Virginia from December 24, 2015, through June 30, 2016. We used a free-text search feature of the electronic medical record to identify patients documented to have the word “hoverboard” in the record. We reported descriptive statistics for patient demographics, types of injuries, body injury location, documented helmet use, injury severity score (ISS), length of stay in the ED, and ED charges.

**Results:**

We identified 83 patients in our study. The average age was 26 years old (18 months to 78 years). Of these patients, 53% were adults; the majority were female (61.4%) and African American (56.6%). The primary cause of injury was falls (91%), with an average ISS of 5.4 (0–10). The majority of injuries were contusions (37.3%) and fractures (36.1%). Pediatric patients tended to have more fractures than adults (46.2% vs 27.3%). Though 20% of patients had head injuries, only one patient reported using a helmet. The mean and median ED charges were $2,292.00 (SD $1,363.64) and $1,808.00, respectively. Head injuries resulted in a significantly higher cost when compared to other injuries; median cost was $2,846.00.

**Conclusion:**

While the overall ISS was low, more pediatric patients suffered fractures compared to adults. Documented helmet use was low, yet 20% of our population had head injuries. Further investigation into proper protective gear and training is warranted.

## INTRODUCTION

Self-balancing personal transporters are increasing in popularity since they were first made available for commercial use in 2001. Previous models, such as the Segway®, had a handle bar for balancing and increased control, yet significant injuries were still reported with the use of these devices.^1^–^4^ Recent hands-free models, commonly referred to as “hoverboards,” have only been available commercially since 2015.^5^ It is estimated that 2.5 million hoverboards have been sold in the U.S., totaling nearly one billion dollars’ worth of sales^6^ and were one of the most popular gifts for Christmas 2015.

The hoverboard is a two-wheeled device that can reach speeds up to 16 miles per hour.^5^ As compared to the Segway®, which contains a sensor in the handlebar for control, each wheel of the hoverboard is responsive to slight movements of each foot independently. This design allows one to move forward, backward, or rotate with only minimal movement of the feet. They are powered by a rechargeable lithium battery. With this new form of travel, there have been emerging guidelines for rider protection, including helmets, knee pads, elbow pads, wrist guards, and shoes,^5^,^8^ but compliance and evidence behind these guidelines are unknown.

With the device’s increasing popularity, reported numbers of injuries related to their use are increasing.^9^–^12^ In addition, there is a risk of the device overheating and subsequent fire hazard^7^ due to a faulty lithium battery.^9^ These problems have resulted in hoverboard recalls, limitations on airplane travel,^7^ or bans from large cities. While there have been several small, single-institution, pediatric-based studies evaluating injury complexes from hoverboard-related injuries,^9^–^12^ to our knowledge no study to date has evaluated the unique injury patterns across all ages associated with its use and the associated healthcare costs. We aimed to address this gap in the literature. The purpose of our study was two-fold: 1) to describe the injury complex associated with hoverboard accidents by examining the types of injuries, areas of the body affected, and differences in pediatric and adult populations; and 2) to examine charges associated with hoverboard injuries within an emergency care setting.

## METHODS

We performed a retrospective chart review on patients with hoverboard-related injuries presenting to local emergency departments (ED) from December 24, 2015, through June 30, 2016. We reviewed patient charts from 10 hospital EDs within an integrated healthcare organization in southeastern Virginia. The total combined volume of these EDs during the study period was 222,611 visits. Each hospital ED uses EPIC as their electronic medical record (EMR) system. The institutional review board at Eastern Virginia Medical School approved this study with a waiver of consent due to its retrospective design.

We identified study patients using a free-text search of the EMR ED documentation provided by emergency nurses and physicians. The terms “hoverboard,” “hover board,” “hoover board,” and “hooverboard” were specified in the search to account for misspellings and typos. We included patients in the study if their ED records matched any of the search criteria during the study period. Patients without a diagnosed injury from a hoverboard were excluded. The data set was reviewed by two emergency physician reviewers (GW and LG) who extracted the discrete data from the EMR using a templated electronic form. The data collection form consisted of 16 discrete questions (i.e., date, location, etc) and two free-text options for descriptions of the mechanism of injury and the injury complex. Out of convenience, the abstractors were not blinded to the hypothesis.

**Population Health Research Capsule**

What do we already know about this issue?

Previous reports on hoverboard-related injuries have focused on the pediatric population and were limited to pediatric EDs.

What was the research question?

We sought to describe the injury complex across all ages and describe the associated healthcare costs in a large community hospital-based system.

What was the major finding of the study?

Pediatric patients suffered more fractures when compared to adults and helmet use was low, yet 20% of our population had head injuries.

How does this improve population health?

Pediatric patients appear to be at risk for injuries related to hoverboards. Further research is needed identify factors associated with the injuries to improve safety standards.

We conducted statistical analysis using IBM SPSS Statistics for Windows, version 22.0.^18^ Descriptive statistics for patient demographics, types of injuries, body injury location, documented helmet use, injury severity score, length of stay (LOS) in the ED, and ED charges were reported. We analyzed data at either the patient or encounter level, depending on the study aim. Patient demographics, injury type, and injury site data were analyzed and reported at the patient level. We analyzed bivariate associations between age category (pediatric or adult) and demographic variables, injury types, and injury sites using Pearson’s chi-square test. Differences in charge amounts between the two age categories were examined using independent samples *t*-test.

For the purpose of analyzing ED costs, data are reported at the encounter level and exclude the two encounters that were admitted to the hospital because we were unable to separate the ED charges from the total charges. Therefore, results from charge data represent 84 encounters. Outliers in charge amounts were addressed by Winsorizing data to the next highest data point within three standard deviations (SD) of the mean.^19^ A test of the assumptions prior to conducting a one-way analysis of variance to identify differences in charges by injury location and injury type revealed heterogeneity across groups; therefore, we used non-parametric tests. The Kruskal-Wallis test was used to examine statistically significant differences in median charges by areas of injury on the body and charges by types of injury. For all statistical tests, we used an alpha level of .05.

## RESULTS

### Data Review

Between December 24, 2015, and June 30, 2016, 84 patients presented to one of the 10 EDs with injuries attributable to hoverboard use. One patient was excluded from the study because she did not sustain injuries from her accident. The remaining 83 patients represent those with diagnosed injuries who were either treated and released from the ED or admitted to an inpatient setting. Of the 83 patients, two presented multiple times to the ED with hoverboard-related injuries, resulting in 86 encounters. These data included two patient encounters that were admitted to the hospital. Both patients were admitted to a medical floor bed and neither required admission to a critical care bed.

### Characteristics of Patients

The majority of patients were female (61.4%) and African American (56.6%), with a mean age of 26.2 years old (standard deviation [SD] =16.20) and median age of 24.0 years old. The youngest patient was 18 months old and the oldest was 78 years old. Additional contextual data taken from notes in the patients’ charts revealed that 14% of patients did not own the hoverboard that led to their injury. The 18-month-old patient was not the primary rider; she fell from a hoverboard while being supported by an older sibling and hit her head on a coffee table. Adult patients (age 18 years and older) constituted over half (53.0%) of the injuries. The mean pediatric age was 11.7 years old (SD = 3.36) and mean adult age was 39.1 years old (SD = 11.35). One-quarter (25.3%) of the patients had one or more comorbidities documented. Comparison of pediatric and adult patients using chi-square analysis found the two groups to be equally distributed in sex, race, and level of comorbidity (Table 1).

### Injury Type and Site

**Figure 1:**
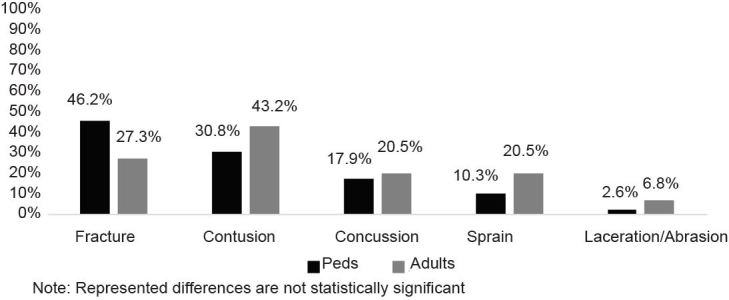
Percent of hoverboard injuries by injury type and age category (*N*=83). *Peds*, pediatrics.

The predominant mechanism of injury was falls (91.6%). Injury severity scores (ISS) ranged between 0 and 10 (M=5.46, SD*=*3.12), indicating low injury complex overall among the patients. The majority of injuries were contusions (37.3%) and fractures (36.1%). Eleven (13.1%) patients suffered from multiple types of injuries, most frequently concussions and contusions. Children most often suffered fractures, whereas adults tended to have contusions (Figure 1).

**Figure 2:**
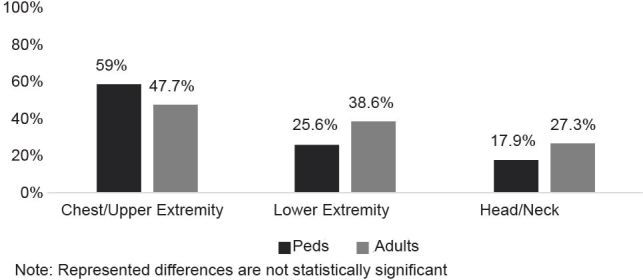
Percent of hoverboard injuries by injury site and age category (N=83). *Peds*, pediatrics.

The location of injury to the body was divided into three zones with respect to distance from the hoverboard: lower extremity, chest and upper extremity, and head and neck. The chest and upper extremity (53.0%) were the most common injury sites, followed by lower extremity injuries (32.5%). Six (7.2%) patients suffered injuries to multiple areas of the body. Both children and adults most frequently suffered injuries to their chest and upper extremity (Figure 2).

To further examine injury type and injury site by age category, we grouped together injuries that fell into more than one category. The relation between these variables was non-significant, χ^2^ (5, N=83) = 7.85, p =.16. The frequency in types of injuries was similar between pediatric and adult patients. Although not statistically different, a higher percentage of pediatric patients sustained fractures compared to adults in the sample (46.2% vs. 27.3%, respectively). The chi-square test of independence revealed no statistically significant difference in injury site by age category, χ^2^ (3, N = 84) = 4.00 *p* =.26. Location of injury was similar between pediatric and adult patients. One patient reported helmet use at the time of the injury, yet 20.2% of patients had a closed head injury.

### Charges from Injuries

The mean charge amount was $2,292.00 (SD=$1,363.64) per ED visit; the median was $1,808.80. The mean charge for an adult patient was $2,532.83 (SD=$1,619.87) and the mean charge for a pediatric patient was $2,014.12 (SD= $935.61). Review of the independent samples *t*-test revealed no statistically significant difference between pediatric and adult patients in overall ED charge amounts, *t(*72.03) = −1.83, *p* = .07. A Kruskal-Wallis test with pairwise comparisons revealed a significant difference in ED charges by injury site, *H* (3) = 8.71, *p* =.03. Patients who sustained head and neck injuries incurred significantly higher charges compared to charges related to lower-extremity injuries (Table 2). No other pairwise comparisons were significantly different in median charge amounts. We conducted a second Kruskal-Wallis test with pairwise comparisons to examine differences in median charges by type of injury. These differences were non-significant, *H (5)* = 10.29, *p* = .07. Tables 3 and 4 provide median charges by injury site and type, respectively. Of note, lacerations and abrasions incurred the highest median charge at $4,800.00 per ED visit; however, data was based on only two encounters.

## DISCUSSION

Our study is the first observational ED-based study to include both pediatric and adult patients in examining the injury complex and charges associated with hoverboard-related injuries. We had a near-equal distribution of pediatric and adult patients in our sample, yet we found that children less than 18 years of age had a higher incidence of fracture than adults. Likewise, both groups were predominately injured by falls. Previous studies have found that children are physiologically at risk for falls, given that they are less mature developmentally in coordination, balance, and motor strength, along with their higher center of gravity. These factors may leave them more susceptible to injuries^13^ compared to their adult counterparts.

There are a limited number of studies on hoverboard injuries that include both adult and pediatric patients. A recent review of hoverboard injuries in the Canadian Hospitals Injury Reporting and Prevention Program (CHIRPP)^12^ found that patients under the age of 19 years were more commonly injured than adults in their case series. However, a noted limitation of the CHIRPP is that it skewed to surveil for pediatric patients. It derives its data from 11 pediatric hospitals and only six general hospitals.^12^ The average age of an injured patient was 12.7 years and only one patient was over the age of 19. Our study sample had a near-equal distribution of adults age 18 years and older (51.8%) and youth (48.2%) seen in the ED. Our study appears to represent a diverse population across 10 different community EDs.^12^

Hoverboard injuries place a patient at increased risk of fractures to the upper extremity according to a pediatric radiology review of fractures related to hoverboards.^10^ These findings were replicated by researchers with CHIRPP, who found that nearly 70% of their injuries occurred in the upper extremity.^12^ In addition, Ho found that 77% of all fractures were to the upper extremity in their sample as well.^11^ Our study had a lower percentage of upper extremity injuries (53.0%) compared to the previously cited literature. Nevertheless, our findings are consistent with prior studies^12^ in which the upper extremities were more often injured compared to either the lower extremity or head and neck.

A key strength of this study is that it is the first to cite financial implications associated with hoverboard injuries. In head-injured patients, the median cost of the hospital care increased by over $1,000.00 compared to non-head injured patients. This rise in cost is most likely due to the cost of CT imaging of the head and cervical spine as compared to radiographs to evaluate extremity injuries.

In our study, the overwhelming cause (92%) of injuries was from falls. Yet, there is currently no formal training on hoverboard use, and recommendations on safety equipment for proper hoverboard use is sparse.^8^ Furthermore, we found that most injuries (30%) occurred to the wrist. Wrist guards have been found to reduce the force from a fall by up to 50% in adults,^14^ but in our study there was no documented wrist brace use.

Similar to our study, documented helmet use in children riding recreational toys is low. Helmet use rate has been documented as low as 8–37% when evaluating children riding non-motorized scooters as compared to our helmet use rate of 1.3%.^15^–^17^ Likewise, the evaluation of head-injured hoverboard patients will increase their ED evaluation by over $1000.00, further highlighting the need for proper protection with helmets.

Although prior studies have demonstrated significant morbidity and mortality associated with collision with motor vehicles,^15^ we did not have any specific cases involving hoverboards colliding with motorized vehicles. However, it is an important consideration when addressing safety concerns, as hoverboards are used on hard surfaces such as sidewalks, parking lots and roads. In addition, there have been concerns over hoverboards catching fire or exploding.^8^ We did not encounter this complication in our population.

Experience is imperative to operating a hoverboard safely. We found that almost 48% of all ED visits for hoverboard-related injuries occurred in the first month after December 24. Likewise, 14% of our patients were on their friend’s or family’s hoverboards and we speculate they were less experienced.

As research on hoverboard injuries increases, differences in injury severity and patient populations with other self-balancing personal transporters are emerging. Compared to recent Segway® injury studies,^2^ our population suffered significantly less severe injuries. We found the average ISS was 5.44 (range 0–10) while the Segway® study reported an ISS range of 4–27 for their 10 admitted patients. They did not provide the ISS for discharged patients. Admitted patients from Segway® injuries suffered severe injury complexes including intracranial hemorrhage, pneumothorax, trimalleolar fracture, pelvic fractures, and complex facial fractures.^2^.

In our review, only four patients required transfer or admission to the hospital for fracture-related care or due to delayed infection caused from a fall. Two pediatric patients required transfer to the local pediatric hospital and two adult patients were admitted from the ED. None required intensive care admission, and most were able to be treated in the ED and safely discharged home. Other differences include our population was significantly younger than Segway®-injured patients and did not have any concomitant anticoagulant use, which may explain why the injuries were less severe.

## LIMITATIONS

Our study has several limitations. First, our sample size does not represent the entire southeastern Virginia population. While it is representative of patients treated in the EDs of one of the primary healthcare systems in the area, our study did not include data from other hospital systems, children’s hospitals, or the large military healthcare system in the area, resulting in possible under-reporting of children and military-based families. Given the small sample size, our study limits its extrapolation to larger populations. Secondly, with our free-text search, it is possible that we did not identify all hoverboard injuries if they used a brand name in documenting. Likewise, we were dependent on the documentation provided in the EMR, and therefore data elements may have been present but not documented, which would have altered our analysis.

The retrospective nature of our study also does not allow us to know the factors surrounding the injuries. For example, we could not confidently identify the speed of the injury or the exact mechanism of the fall that resulted in the injury complex. Only two reviewers (GW and LG) extracted data and each then reviewed the other’s work to ensure accuracy. However, no inter-rater reliability score was examined. Finally, the sample size may have limited the study’s power to detect statistical differences between children and adults in the types of injuries sustained, areas of the body affected, and ED charge amounts. With respect to our cost analysis, we had a small sample size for lacerations and concussions, which limited the comparative value of the cost.

Follow-up studies with at least a full year’s data are warranted to increase statistical power and to fully explore seasonality in injury patterns. The fact that our study did not have a majority youth representation may be because we did not have access to data from the local pediatric hospital.

## CONCLUSION

While the overall ISS of hoverboard-related injuries was low, children less than 18 years of age had a higher percentage of fractures compared to their adult counterparts. Documented helmet use in the current study was extremely low, with 20% of patients experiencing closed head injuries, leading to an increased cost for those ED visits. Further investigation into the risk of hoverboard use is needed. Prospective studies are needed to identify the factors associated with hoverboard-related injuries that will serve to better inform safety standards in using protective equipment.

## Figures and Tables

**Table 1:** Characteristics of patients with hoverboard injuries (N=83).

	Pediatric (n=39); n (%)	Adult (n=44); n (%)	χ^2^	p-value
Sex				
Female (n=51)	24 (47.1)	27 (52.9)	.00	.99
Male	15 (46.9)	17 (53.1)		
Race				
African-American (n=47)	18 (38.3)	29 (61.7)	3.29	.07
White (n=36)	21 (58.3)	15 (41.7)		
Comorbidities				
None (n=62)	33 (53.2)	29 (46.8)	3.83	.05
1 or more (n=21)	6 (28.6)	15 (71.4)		

* p < .05.

**Table 2:** Results of Kruskal-Wallis pairwise comparisons of charges by injury site (N=84).

Pairwise comparisons	Test statistic	Standard error	Standard test statistic	p-value
Lower extremity-head/neck*	22.17	7.88	2.82	.03
Lower extremity-chest/upper extremity	12.95	6.50	1.99	.28
Multiple-chest/upper extremity	8.46	10.70	.79	1.00
Multiple-head/neck	17.69	11.58	1.53	.76
Chest/upper extremity-head/neck	9.23	7.09	1.30	1.00
Lower extremity-multiple	−4.49	11.23	−.40	1.00

* p < .05.

**Table 3:** Median emergency department hoverboard-injury charges by site (N=84).

Injury site on body	N	Median ED Charge ($)
Head/neck	17	2,846
Chest/upper extremity	39	1,873
Lower extremity	22	1,289
Multiple sites	6	1,802
Overall	84	1,809

*ED*, emergency department.

Note: Medians are based on adjusted/Winsorized charge values.

**Table 4:** Median emergency department hoverboard-injury charges by type (N=84).

Injury site on body	N	Median ED charge ($)
Laceration/abrasion	2	4,810
Concussion	8	2,847
Fracture	29	1,892
Contusion	23	1,672
Sprain	11	1,591
Multiple types	11	2,047
Overall	84	1,8089

*ED*, emergency department.

Note: Medians are based on adjusted/Winsorized charge values.
